# Rasd1 Modulates the Coactivator Function of NonO in the Cyclic AMP Pathway

**DOI:** 10.1371/journal.pone.0024401

**Published:** 2011-09-07

**Authors:** Shufen Angeline Ong, Jen Jen Tan, Wai Loon Tew, Ken-Shiung Chen

**Affiliations:** 1 School of Biological Sciences, Department of Genomics and Genetics, Nanyang Technological University, Singapore, Singapore; 2 Agency for Science, Technology and Research, Singapore, Singapore; St. Jude Children's Research Hospital, United States of America

## Abstract

All living organisms exhibit autonomous daily physiological and behavioural rhythms to help them synchronize with the environment. Entrainment of circadian rhythm is achieved via activation of cyclic AMP (cAMP) and mitogen-activated protein kinase signaling pathways. NonO (p54nrb) is a multifunctional protein involved in transcriptional activation of the cAMP pathway and is involved in circadian rhythm control. Rasd1 is a monomeric G protein implicated to play a pivotal role in potentiating both photic and nonphotic responses of the circadian rhythm. In this study, we have identified and validated NonO as an interacting partner of Rasd1 via affinity pulldown, co-immunoprecipitation and indirect immunofluorescence studies. The GTP-hydrolysis activity of Rasd1 is required for the functional interaction. Functional interaction of Rasd1-NonO in the cAMP pathway was investigated via reporter gene assays, chromatin immunoprecipitation and gene knockdown. We showed that Rasd1 and NonO interact at the CRE-site of specific target genes. These findings reveal a novel mechanism by which the coregulator activity of NonO can be modulated.

## Introduction

The cAMP-dependent pathway is known to respond to information obtained from numerous extracellular stimuli to regulate processes including synaptic plasticity, neuronal differentiation, circadian rhythm, memory, and glucose homeostasis [1], [2], [3], [4], [5], [6]. Despite the involvement of unique neurotransmitters, hormones or other signals, and different intracellular signaling systems, these pathways all converge at the nucleus. Hence, specificity of the signal and the pathway induced is crucial to ensure that specific proteins are transcribed to perform precise functions in a tissue- and/or temporal-specific manner. This specificity is achieved by the type of signals, how the signals are detected and relayed to specific signaling proteins responding to the stimuli, and the subsequent interactions with other proteins, and is dependent on cell type and contexts. Regulation of the pathway can occur at any step of the signal transduction process but one of the more prominent regulations is at the transcriptional level. Regulation of the pathway at the transcriptional level is achieved by various mechanisms including inhibition of core transcription factor activity, sequestration, and competition for limiting factor [7], [8], [9].

NonO is predominantly localized in the paraspeckles [10], a sub-compartment of the nucleus, and is a member of the family of RNA-Recognition Motif (RRM) containing proteins [11]. NonO is a co-activator of CREB and has been known to serve in both transcriptional activation and repression [12], [13], [14], [15]. In our current study, NonO is identified as a binding partner of Rasd1, a monomeric G protein belonging to the RAS family [16], [17]. Traditionally, RAS proteins function as cytoplasmic signal transducers of diverse intracellular signaling pathways including the cAMP-dependent pathway [16]. Similar to its other family members, Rasd1 harbours a CAAX motif at its C-terminal and displays a high degree of conservation in its G boxes, which are responsible for the guanine nucleotide binding and hydrolysis activities of RAS proteins. Mutations in the G boxes have been shown to disrupt the functions of RAS proteins [16], [18], [19], [20], [21], [22], [23], [24]. Rasd1 has been shown in various studies to be involved as signal transducers of multiple signaling pathways, including iron homeostasis, growth hormone secretion and circadian rhythm [20], [25], [26], [27], [28], [29]. Recently, Rasd1 has also been observed to reside in the nucleus, serving as a transcriptional repressor of glycogen synthase kinase 3β [19] as well as an inhibitor of the cAMP-dependent pathway [20], [28], [29].

In this study, we identify NonO as a novel binding partner of Rasd1. This is the first study that shows the novel interaction of a RRM-possessing protein with a monomeric G protein. In the nucleus, Rasd1 binds to NonO and regulates the cAMP-dependent pathway at the transcriptional level. GTP-hydrolysis activity of Rasd1 is required for repressing CREB activity. We propose a new mechanism of regulating the cAMP-dependent pathway at the transcriptional level via modulation of the co-activator's function. Binding of Rasd1 to NonO modulates NonO's functions by changing NonO from a co-activator to a co-repressor of the cAMP-dependent pathway. Rasd1 and NonO cooperate to suppress the transcription of a subset of CRE-containing genes, *NR4A 1* & *2*. This finding adds weight to how specificity of signaling pathways is achieved via the usage of different interacting partners to modulate the function of a multi-tasking co-regulator [30].

## Results

### NonO was identified as a novel interacting partner of Rasd1 via affinity pull-down assay followed by mass spectrometry analysis

**Figure 1:**
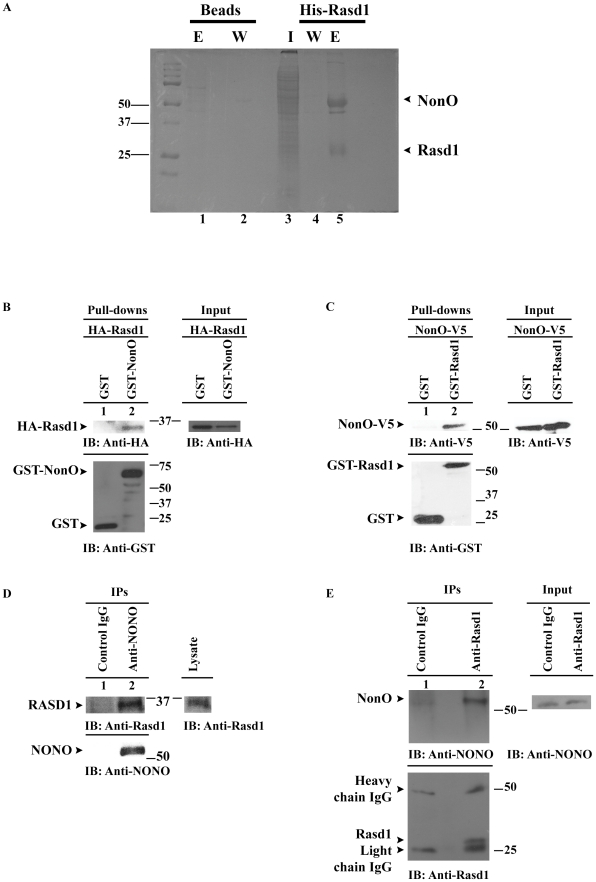
NonO is identified as a novel interacting partner of Rasd1 via affinity pull-down assay. (A) Coomassie blue stained SDS-PAGE gel of affinity pulldown assay. Ni-NTA magnetic beads were incubated either with lysate from pHis-Rasd1 transfected COS-7 cells or with lysate of empty vector transfected cells. Next, washes were conducted to remove non-specific binding proteins. His-Rasd1 bound to the magnetic beads was then incubated with PC-12 lysate. The beads were boiled to separate the protein complexes for fractionation on SDS-PAGE (12%). The 55 kDa, 50 kDa and 30 kDa bands were observed in the elute lane of His-Rasd1 (Lane 5) but not in the elute lane of the negative control (Lane 1). Protein bands were excised for further analysis using mass spectrometry. The proteins identified were NonO, Tubulin beta 5 and Rasd1, respectively. E, elute; W, wash; and I, input. (B) Co-precipitation assay was performed to study *in vivo* interaction between Rasd1 and NonO. COS-7 cells were co-transfected with plasmids expressing HA-Rasd1 and either GST-NonO or GST. The lysates were then incubated with GSH-linked magnetic beads to precipitate GST-tagged proteins. HA-Rasd1 was observed to co-precipitate specifically with GST-NonO but not GST (compare Lanes 1 with 2). (C) A similar interaction assay was performed for NonO-V5 and GST-Rasd1 proteins. In this experiment, COS-7 cells were co-transfected with pNonO-V5 and either pGST-Rasd1 or pXJGST. GSH-linked magnetic beads were added to the cell lysates to pull-down GST-tagged proteins. NonO-V5 was observed to be co-precipitated with GST-Rasd1 but not with GST (compare Lanes 1 with 2). (D) To study the *in vivo* interaction of endogenous Rasd1 and NonO, co-IP was performed on HEK293T cell lysates incubated with either rabbit anti-NonO or rabbit control IgG. Detection of the blot with anti-Rasd1 showed that RASD1 was co-IP specifically by NONO (Lane 2). (E) A similar Co-IP was conducted using mouse brain lysate incubated with either anti-Rasd1 or goat control IgG. NonO was only co-precipitated by lysate incubated with anti-Rasd1 (Lane 2). NonO-V5 is detected with anti-V5 (Invitrogen, USA, CA); GST-tagged proteins are detected with anti-GST (Santa Cruz, USA, CA); HA-Rasd1 is detected with anti-Xpress (Invitrogen, USA, CA); endogenous NonO is detected with goat anti-NONO; and endogenous Rasd1 is detected with goat anti-Rasd1.

To facilitate our understanding of Rasd1's physiological functions, an *in vitro* affinity assay was performed to identify novel interacting partners of Rasd1. COS-7 cells were used to over-express His-Rasd1 for subsequent interaction studies. The His-tagged proteins were then purified by Ni-NTA magnetic beads. This was followed by incubation with cell lysate extracted from PC-12 cells, which are known to express endogenous Rasd1 [26]. The complexes bound to Rasd1 were eluted and fractionated by SDS-PAGE. Three distinct bands were observed on the elute lane of beads bound with His-Rasd1 but not in the elute lane of negative control (compare Figure 1A, Lanes 1 with 5). These bands were excised, and mass spectrometry was conducted to determine the identity of the proteins. The band that was approximately 30 kDa on the gel (Figure 1A, Lane 5) was identified to be Rasd1. It was logical that the band was present in the elute lane for cells transfected with pHis-Rasd1 but not in the negative control. The next two bands at approximately 50 kDa and 55 kDa on Lane 5 were analysed with mass spectrometry as well. The bands were identified as Tubb5 and NonO, respectively (Figure 1A, Lane 5). In the present study, we focused exclusively on investigating the interaction between NonO and Rasd1.

### *In vivo* interaction study confirms NonO as a novel binding partner of Rasd1

To validate the interaction between Rasd1 and NonO, an *in vivo* interaction study was conducted by co-transfecting COS-7 cells with plasmids expressing either HA-Rasd1 and GST-NonO or HA-Rasd1 and GST. GST and GST-tagged proteins were purified with MagneGST™ particles. Bound complexes were eluted and then fractionated on SDS-PAGE, followed by western blot. GST-NonO was observed to co-precipitate HA-Rasd1 specifically (Figure 1B, Lane 2). A similar observation was observed when cells were co-transfected with pGST-Rasd1 and pNonO-V5. In this case, GST-Rasd1 was purified using GSH-linked beads, and NonO-V5 was co-precipitated along with GST-Rasd1 (Figure 1C, Lane 2). Previous studies have shown that Rasd1 and NonO are expressed in HEK293T cells [13], [26]; hence co-IP was carried out using HEK293T cell lysates. An antibody against NONO was used to precipitate endogenous NONO, and RASD1 was observed to be co-precipitated with NONO (Figure 1D, Lane 2). In addition, co-IP using mouse brain lysate was also performed. Rasd1 was purified by anti-Rasd1 and NonO was observed to co-purify specifically with Rasd1 (Figure 1E, Lane 2). The results obtained from co-precipitation and co-IP assays show that interaction of Rasd1 and NonO is specific and conserved across species (mouse and human).

### Rasd1 interacts with NonO to suppress CREB-mediated transcription

**Figure 2:**
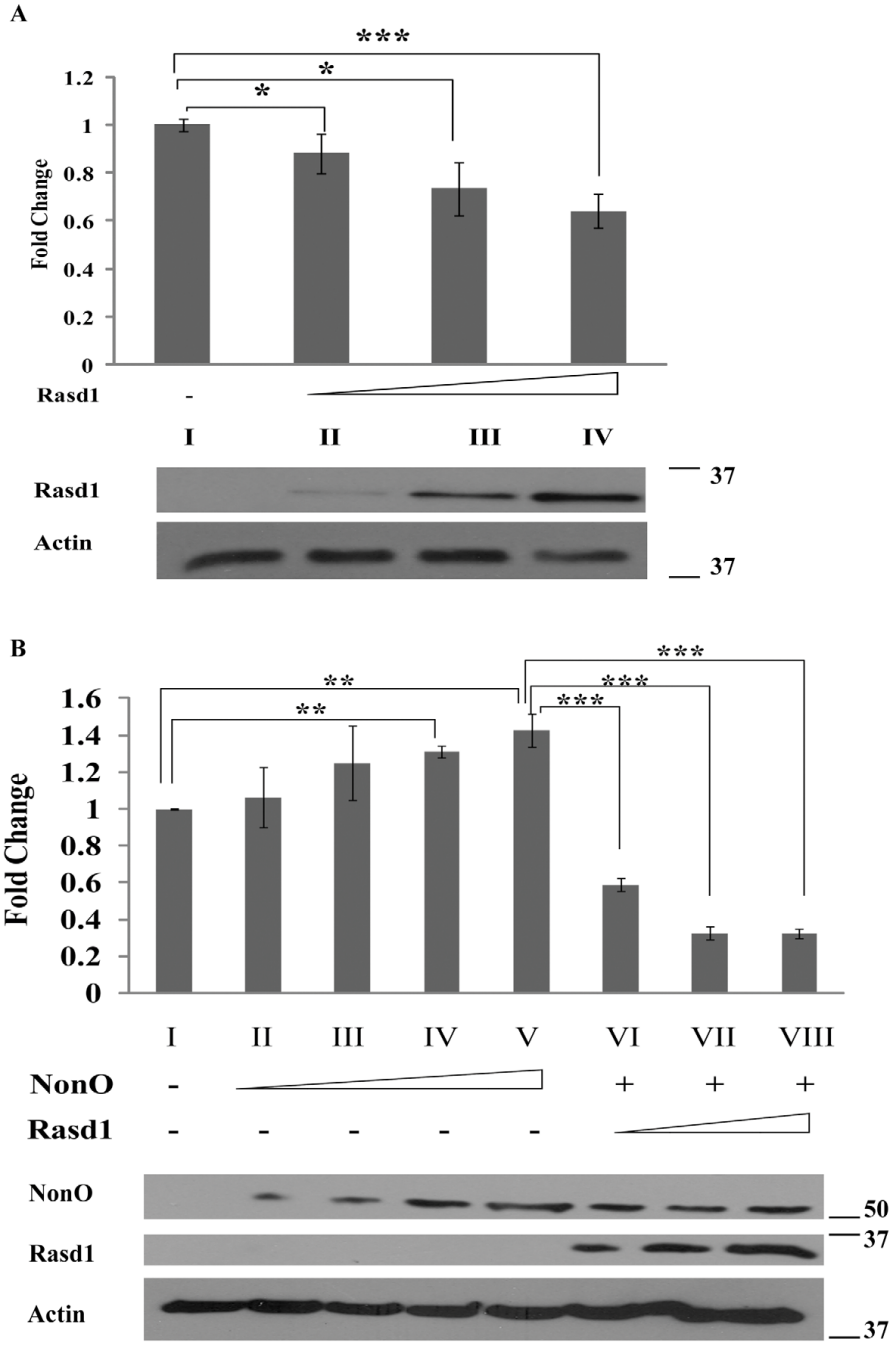
Effects of Rasd1 and NonO in the cAMP-signaling pathway in HEK293T cells were studied using reporter gene assay. (A) NonO- (2 µg) and Rasd1- (or Rasd1 mutants) (2 µg) expressing plasmids were co-transfected in HEK293T cells. Immunofluorescence was performed 2 days after transfection. Cells were transfected with different amounts of His-Rasd1-expressing vector (0, 0.5, 1 and 2 µg) along with reporter vector and CREB-expression vector. Two days later, cells were induced with forskolin (20 µM) for 4 hours before harvest. Luciferase assays were subsequently performed on the cell lysates. The results show that luciferase expression was reduced by 40% upon transfection of pHis-Rasd1 (compare bars I and IV). The suppression of luciferase expression by Rasd1 is dosage-dependent, as increasing amounts of pHis-Rasd1 resulted in further down-regulation of luciferase expression (compare bars II and III; and bars III and IV). (B) Parallel experiments were conducted by transfecting different amounts of pNonO-V5 (0, 0.1, 0.2, 1 and 2 µg) into HEK293T cells. The presence of NonO in HEK293T cells results in a slight up-regulation of luciferase expression of up to 130% (compare bars I and V), and this up-regulation is dosage-dependent (Compare bars II and IV; bars II and V). Next, pNonO-V5 and pHis-Rasd1 were co-transfected in HEK293T cells to determine if the proteins cooperate to influence CREB's transactivation functions. Different amounts of pHis-Rasd1 (0, 0.5, 1 and 2 µg) were co-transfected with pNonO-V5 (2 µg), and cells were treated with forskolin before harvest. We observed that luciferase expression was reduced by 80% in the presence of both NonO and Rasd1 (compare bars I and VIII). The activation effect observed when NonO was transfected alone was abolished upon co-transfection of NonO with Rasd1 in the cells (compare bars V with VIII). The suppressive effect on luciferase expression was further enhanced in the presence of NonO and Rasd1 as compared to that of transfection of Rasd1 alone (compare (A) bar IV and (B) bar VIII). Representative Western blots were included to show the protein expressions of Rasd1 and NonO. Actin was included as a loading control. Rasd1 is detected with anti-Xpress; NonO is detected with anti-V5; and actin is detected with anti-actin.

Rasd1 protein has been shown to play multiple roles in the regulation of the cAMP pathway, including heterologous sensitisation of adenylyl cyclase 1 via G_βγ_, attenuation of cAMP-stimulated hGH secretion, and inhibition of adenylyl cyclase through G_iα_ in the HEK293T cell line [20], [28], [29]. Similar involvement was shown for NonO in the cAMP pathway. NonO interacts with TORC2 (transducer of regulated CREB-binding proteins 2) and functions as a co-activator to upregulate transcription of *NR4A2* and *FOS* upon activation of the cAMP-dependent pathway; it has also been shown to be involved in regulation of *CYP17*′s transcription via this pathway [13], [14]. In this paper, we employed the PathDetect CREB trans-Reporting System to investigate the effects of Rasd1 and NonO on CREB-mediated gene transcription in HEK293T cells. Cells were transfected with pHis-Rasd1 along with luciferase reporter gene driven by CREB-responsive promoter and CREB-expressing vector. Prior to harvest, cells were induced with forskolin for 4 hours to study the effect of Rasd1 on the cAMP pathway. We observed that Rasd1 repressed the CREB-mediated transcription in a dose-dependent manner (compare Figure 2A, bars I with II–IV), supporting the findings reported in the previous study [19]. We transfected plasmid-expressing NonO in HEK293T cells to observe the effect of NonO on CREB's activity via reporter gene assay. Transfection of NonO in the cells only led to a mild activation of the CREB-luciferase reported activity (compare Figure 2B, bars I with V).

When plasmids expressing NonO and Rasd1 were co-transfected in HEK293T cells to study the effect of these proteins on the pathway, the CREB-luciferase reporter activity was reduced by 80% (compare Figure 2B, bars I and VIII). The up-regulation effect of NonO on the pathway was also abolished in the presence of Rasd1 (compare Figure 2B, bars V with VIII). In addition, the repressive effect of Rasd1 on the pathway was enhanced in the presence of NonO (compare Figure 2A, bar IV with Figure 2B, bar VIII). These results suggest that Rasd1 acts as a regulator of NonO to modulate its function in transcription.

### Rasd1 and NonO co-localise in the nucleus

**Figure 3:**
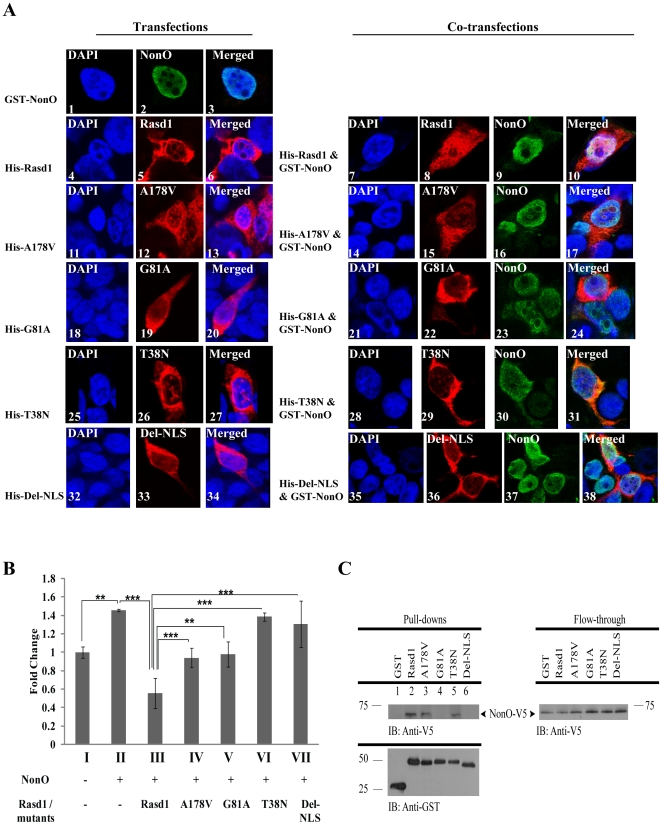
GTP hydrolysis activity of Rasd1 is required to cooperate with NonO to suppress CREB's activity. (A) NonO localises primarily in the nucleus of HEK293T cells transiently transfected with pNonO-V5 (2 µg) (Figure A2). Rasd1 is distributed throughout the cell in the event of individual transfection of pHis-Rasd1 (2 µg) (Figure A5). A considerable increase in the amount of Rasd1 was observed to be present in the nucleus upon the event of co-transfection with pGST-NonO (compare Figures A5 with A8). The sub-cellular location of NonO was unaffected in cells co-transfected with pHis-Rasd1 (compare Figures A2 with A9). Rasd1 mutants, A178V, G81A, T38N and Del-NLS, display similar cellular distribution to Rasd1 in the event of single transfection (Figures A 5, 12, 19, 26, and 33). However, co-transfection of plasmids expressing Rasd1 mutants and NonO did not affect the mutants' sub-cellular distribution, unlike that of wild-type Rasd1 (compare Figures A5 with A 15, 22, 29, and 36). Likewise, the sub-cellular distribution of NonO was also unaffected by the presence of Rasd1 mutants, A178V, G81A and Del-NLS (compare Figures A2 with A 16, 23, and 37). Interestingly, NonO was translocated to the cytoplasm in the presence of T38N (compare Figures A2 with A30). (B) Effects of Rasd1 mutants on the CREB pathway in the presence of NonO were studied in HEK293T cells. Cells were co-transfected with pNonO-V5 and either Rasd1 or Rasd1 mutants, and luciferase assays were performed after lysis of cells. Cells were induced with forskolin for 4 hours prior to harvesting. The CREB-mediated transcription was repressed in cells co-transfected with pNonO-V5 (2 µg) and pHis-Rasd1 (2 µg) (Compare Bars II with III). However, the repressive effect on CREB is abolished in cells transfected with Rasd1 mutants expressing plasmids compared with cells transfected with pHis-Rasd1 (Compare Bars III with IV–VII). ‘*’ – p<0.05; ‘**’ – p<0.01; ‘***’ – p<0.001. (C) Interaction studies of NonO and Rasd1 mutants were studied via co-transfection of pNonO-V5 and pGST-Rasd1 mutant clones in COS-7 cells. GST-pulldown was subsequently performed and similar to wild-type, only constructs T38N and A178V were able to interact with NonO (Lanes 2, 3 and 5).

Co-localisation studies were performed by co-transfection of Rasd1- and NonO- expressing plasmids to study if Rasd1 and NonO influence each other's sub-cellular localisation. Consistent with previous reports, we observed that in cells transfected with pNonO-V5, NonO mainly resides in the nucleus (Figure 3, A2) [12], [13], [14], [15], [31], [32], [33]. In addition, the localization of NonO was not affected by the presence of Rasd1 (Figures 3, A2 & A9). On the other hand, Rasd1 was distributed throughout the cells transfected with pHis-Rasd1 (Figure 3, A5), which is consistent with previous reports [19], [34]. In the event of co-transfection of pGST-NonO and pHis-Rasd1, a substantial increase in the nuclear localisation of Rasd1 was observed when compared with transfection with Rasd1 expression vector alone (compare Figures 3, A5 and A8). The finding suggests that nuclear presence of Rasd1 is enhanced by NonO.

### GTP hydrolysis activity of Rasd1 is required for repression of CREB-mediated transcription

As a member of the monomeric G protein family, Rasd1 possesses GTP binding and hydrolysis activity. RAS proteins are activated when GTP-bound and inactivated when GDP-bound. In order to obtain a better understanding of the mechanism by which the cooperation of Rasd1 with NonO mediates a suppressive effect, three Rasd1 mutants with point mutations of the conserved residues at the G boxes of RAS proteins were constructed. The mutants consist of two constitutively active mutants of Rasd1 – A178V and G81A – and an inactive mutant, T38N. Similar Rasd1 mutants have been constructed, including H-Ras (H-Ras[A146V] and H-Ras[G60A]), and Rab11 (Rab11[S25N] and Rab11[Q70L]) [21], [22], [34], [35]. The A178V mutation is expected to interrupt the guanyl nucleotide-binding pocket, resulting in an enhanced exchange rate of guanine nucleotides [20], [35]. Since, guanyl nucleotide exchange is the rate limiting step in the activation of G proteins, and the intracellular levels of GTP is higher than GDP, an increase in the nucleotide exchange rate is supposed to lead to an increased occupancy in the active GTP-bound state. Hence, the mutants bind a higher proportion of GTP to GDP *in vivo* and behave functionally as constitutively active signal transducers [20], [35]. However, the mutant has an overall lower activity than the wild type. G81A, another constitutively active mutant of Rasd1, carries a point mutation in the G3 box guanine residue. This mutation in H-Ras has been shown to interfere with its interaction with GTPase-activating proteins (GAPs), thus leading to a protein that is consistently bound to GTP [21]. In the T38N mutant, the key residue in the G2 box is switched from threonine to asparagine. This mutation is known to severely reduce the binding affinity of Rab11 to GTP, leaving the GDP-binding properties unchanged [22]. Therefore, the mutation results in an inactive Rasd1 that is constantly bound to GDP. In a previous study [19], it was suggested that a putative nuclear translocation signal (NLS) on the C-terminal of Rasd1 permitted its nuclear translocation. Hence, we constructed a Rasd1 mutant, Del-NLS, in which the putative nuclear localisation signal was deleted, to further understand how increased nuclear presence of Rasd1 occurs in the company of NonO.

We observed from our immunofluorescence study that, although all mutants (Figure 3A, 12, 19, 26 and 33) displayed similar sub-cellular distribution as wild-type Rasd1 (Figure 3A, 5), none of them displayed an increase in nuclear distribution upon co-transfection with pGST-NonO (Figures 3A: 15, 22, 29, and 36). Interestingly, NonO was observed in the cytoplasm upon co-transfection with T38N (Figure 3A, 30), unlike all other co-transfections where the sub-cellular location of NonO was observed primarily in the nucleus (Figures 3A, 9, 16, 23, and 37).

Next, we compared the ability of wild-type and mutant Rasd1 to suppress CREB-mediated transcriptional activity in HEK293T cells overexpressing NonO. We observed that none of the Rasd1 mutants were able to effectively suppress CREB-mediated transcriptional activity when compared to the wild-type Rasd1 (compare Figure 3B, bars III with IV–VII). Interaction studies were subsequently performed, and the results show that mutants G81A and Del-NLS were unable to interact with NonO (Figure 3C, lanes 4 and 6); however, A178V and T38N were still able to interact with NonO (Figure 3C, lanes 3 and 5), and a substantial amount of NonO was present in the cytoplasm (Figure 3C, 38) in the presence of T38N. Taken together our findings suggest that interaction between NonO and wild-type Rasd1 is required for the suppression of CREB-mediated transcription.

### Rasd1 cooperates with full-length NonO to repress CREB activity

**Figure 4:**
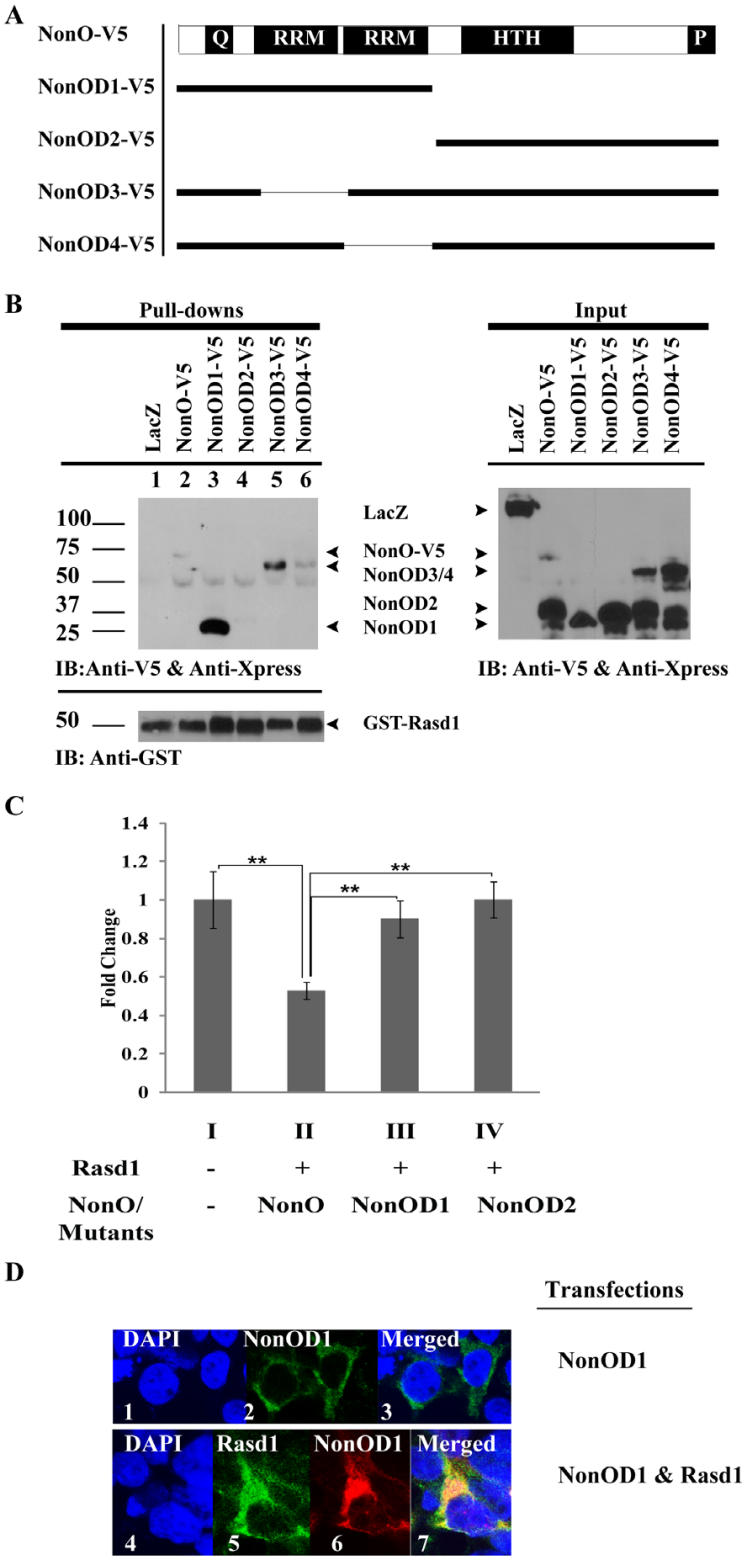
Rasd1 requires full-length NonO to suppress the cAMP pathway in HEK293T cells. (A) Schematic drawing of the locations of specific domains of NonO protein and its truncated constructs. Q, glutamine-rich region; RRM, RNA-recognition motif; HTH, helix-turn-helix and highly-charged region; P, proline-rich region. Bipartite NLS is located within HTH. (B) HEK293T cells were transfected with pGST-Rasd1 along with various constructs of NonO-V5. Lysates were then incubated with MagneGST™ particles, which enable binding of GST-Rasd1. Only NonOD2-V5 did not interact with GST-Rasd1 (Lane 4). Anti-V5 and Anti-Xpress were used for detection of LacZ and all truncated clones of NonO; anti-GST was used for detection of GST-Rasd1. (C) Rasd1 (2 µg) was co-transfected with either NonO or NonO mutants (2 µg) and luciferase assay was performed subsequently. Neither mutant was able to repress CREB's activity in the presence of Rasd1 unlike that of wild-type NonO (compare Histograms II with III and IV). (D) Immunofluorescence studies of NonOD1 and Rasd1 in HEK293T cells. GST-NonOD1 is primarily localised in the cytoplasm (Figure D2). In the event of co-transfection with GST-NonOD1, His-Rasd1 is localised in the cytoplasm, whereas the sub-cellular distribution of His-Rasd1 was concentrated in the nucleus in the presence of NonO (compare Figure 3 A8 with Figure 4 D5).

To map the interaction domain between Rasd1 and NonO, a series of truncated NonO were constructed to determine the site at which Rasd1 binds (Figure 4A). HEK293T cells were co-transfected with pGST-Rasd1 and various plasmids containing truncated NonO. GST-pulldown was performed using cell lysate prepared from HEK293T cells transfected with the respective plasmids. All truncated constructs except NonOD2 were able to interact with Rasd1 (Figure 4B). Unlike other constructs which possessed at least one RRM domain, there was no RRM domain present in NonOD2. The results suggest that one RRM domain is sufficient for interaction with Rasd1. Next, pGST-Rasd1 was co-transfected with either NonOD1- or NonOD2-expressing plasmids to determine if the RRM domains (NonOD1) are adequate for cooperation with Rasd1 to repress CREB's activity. We observed that, unlike wild-type NonO, neither truncated clones were able to repress CREB's activity in the presence of Rasd1 (compare Figure 4C, bars II with III and IV). This implies that both the RRMs and the DNA-binding region of NonO were required for functional interaction between Rasd1 and NonO. Next, we investigated if the RRM domains of NonO are responsible for the increased nuclear distribution of Rasd1 in the presence of NonO via immunofluorescence studies. The NonOD1 construct lacking the nuclear localisation motif was unable to translocate into the nucleus (Figure 4D2). In the event of co-transfection of NonOD1 with Rasd1, Rasd1 was present only in the cytoplasm, which is different in comparison to co-transfection of NonO with Rasd1 (compare Figure 3C2 with Figure 4D5). The results suggest that NonO may play a role in retaining Rasd1 in the nucleus and that the nuclear presence of both NonO and Rasd1 is required for down-regulating CREB-mediated transcriptional activity (Figure 4C, bar III).

### *NR4A1* and *NR4A2* are target genes regulated by both Rasd1 and NonO in the cAMP pathway

Expression of both Rasd1 and NonO leads to the repression of CREB-mediated transcriptional activity (Figure 2B, bar VIII). To identify endogenous genes regulated by the combined actions of both Rasd1 and NonO, plasmids expressing Rasd1 and NonO were co-transfected into HEK293T cells. This was followed by quantitative Real time PCR to study the transcriptional activity of endogenous genes regulated by the cAMP pathway.

Several endogenous genes known to have a functional CRE site were studied. This includes *PER1 (Period1)*, *CYP17*, *NR4A1*, *NR4A2*, *NR4A3*, *FOS*, and *Prolyl-4-hydroxylase α 1 (Prolyl α)*. However, out of all the genes studied in HEK293 cells, only the transcript levels of *NR4A1*, *NR4A2* and *FOS* were consistently induced by forskolin (Figure S1). It has previously been shown that not all genes that contain CRE elements (such as *PEPCK*, *BDNF*, and *insulin*) are regulated by CREB in PC-12 cells [36]. Hence, our results support the finding that CREB regulates only a certain cohort of CRE-containing promoters in different cell lines, perhaps due to a difference in the expression of co-regulators. Therefore, in subsequent studies conducted with HEK293T cells, we focus on the effect of NonO and Rasd1 on the transcription of the endogenous *NR4A1*, *NR4A2* and *FOS*.

**Figure 5:**
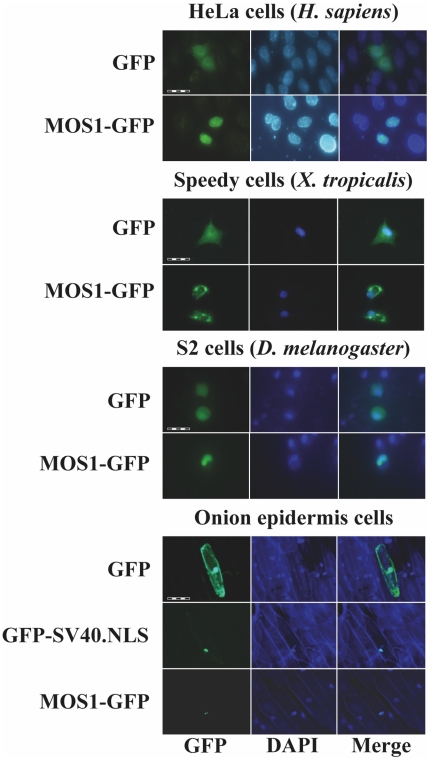
Rasd1 and NonO interact at the CRE-site of the target promoter to repress the transcription of endogenous cAMP target genes, *NR4A1* and *NR4A2*, but not *FOS*. (A) Quantitative real-time study was performed to study the effect of over-expression of Rasd1 and NonO on endogenous CREB-target genes in HEK293T cells. Induction of the cAMP pathway with forskolin (20 µM for 45 minutes) leads to the up-regulation of cAMP target genes – *NR4A1*, *NR4A2* and *FOS* (compare the respective Bars I and II of each gene). Co-transfection of plasmids expressing Rasd1 and NonO in forskolin-induced HEK293T cells leads to the down-regulation of *NR4A1* and *NR4A2* transcripts (compare the respective Bars II and IV of each gene). The expression of *FOS* transcript was not affected by the presence of Rasd1 (compare Bars II and IV of *FOS*). Expression of *YWHAH* transcript was not affected either by treatment of cells with forskolin or in the presence of Rasd1 and NonO, and was shown as a negative control (Bars I–IV of *YWHAH*). β-actin was used as an internal control for normalization. On the right are representative western blots showing expression of transfected NonO and Rasd1 in HEK293T cells. Actin serves as a loading control. NonO is detected by mouse anti-V5; Rasd1 is detected with mouse anti-GST; and actin is detected with anti-actin. (B) Quantitative real-time study of the effect of Rasd1 and NonO on endogenous genes of NONO-knockdown HEK293T cells. Similar to (A), induction of cells with forskolin leads to the up-regulation of cAMP target genes – *NR4A1*, *NR4A2* and *FOS* (compare Bars I with II of each gene). Next, rescue of NONO was performed by transfection of plasmid expressing NonO in *NONO*-knockdown cells. The transcripts of *NR4A1*, *NR4A2* and *FOS* were up-regulated, confirming the involvement of NonO in the regulation of their transcription (compare Bars III with IV of each gene). In *NONO*-knockdown cells with over-expression of Rasd1, the transcripts levels of *NR4A1*, *NR4A2* and *FOS* were comparable to *NONO*-knockdown cells (compare bars III and V of each gene). This suggests that Rasd1 requires NONO to repress the transcription of the CREB-target genes. Repression of target genes, *NR4A1* and *NR4A2*, was observed in NONO-knockdown cells that were co-transfected with Rasd1 and NonO (compare Bars IV with VI of each gene). The expression of *FOS* transcript remained unaffected by the presence of Rasd1 (compare Bars IV with VI of *FOS*), similar to that in Bar IV of (A). β-actin was used as an internal control for normalization. On the right are representative western blots showing expression of NonO and Rasd1in HEK293T cells. Actin serves as a loading control. NonO and endogenous NONO are detected by anti-NONO; Rasd1 is detected with anti-GST; and actin is detected with anti-actin. (C) ChIP was performed using forskolin-treated (20 µM for 15 minutes) HEK293T cell lysates transfected with either pGST-Rasd1 or pGST (negative control) and incubated with either no antibody control (No AB) or anti-NONO (NONO). Primers targeting the CRE-site of *FOS* and *NR4A2* promoters were used for subsequent PCR study. The results indicated that in the presence of Rasd1, more NONO was bound to the *NR4A2* promoter (compare Lanes 2 and 3). The amount of NONO bound to the *FOS* promoter displayed no significant difference with or without transfection of pGST-Rasd1 (Lanes 5 and 6). I, input; and E, elute. (D) Next ChIP was performed similar to (C) using forskolin-treated HEK293T cells transfected with His-Rasd1 (Rasd1), empty vector (Vec), or His-Del-NLS (Del-NLS; mutant Rasd1 that does not interact with NonO). The sonicated lysates were subsequently incubated with Anti-Xpress, and primers targeting the CRE-sites on the *FOS* and *NR4A2* promoters were used for PCR study. Results indicate that neither Rasd1 nor mutant Rasd1 binds to the *FOS* promoter (Lanes 5 and 6). On the other hand, Rasd1 but not its mutant, Del-NLS, specifically binds to the CRE-site of the *NR4A2* promoter, suggesting that interaction of Rasd1 and NonO is required to suppress the transcription of *NR4A2* (compare Lanes 2 and 3). I, input; and E, elute.

We observed that induction of *NR4A1* and *NR4A2* gene expression by forskolin was abolished upon co-transfection of pNonO-V5 and pGST-Rasd1 (compare Figure 5A, bars II and IV, for the respective genes). However, the induction of *FOS* gene expression by forskolin was not affected by the presence of NonO and Rasd1 (compare Figure 5A, bars IV of *FOS*, *NR4A1* and *NR4A2*). These results suggest that Rasd1 and NonO regulate a subset of the CREB target genes in HEK293T cells. The expression of negative control *YWHAH* was not affected by Rasd1 and NonO and/or forskolin (Figure 5A, bars I–IV).

To determine if Rasd1 is able to regulate transcription of CREB target genes on its own, we performed experiments to knockdown endogenous *NONO* in HEK293T cells. *NONO-*knockdown was able to reduce the transcriptional activity of CREB target genes to levels comparable to that observed in uninduced HEK293 cells (compare Figure 5B, bars I with III of each gene). Transcription levels of these genes were restored to levels similar to that in cells induced with forskolin alone when *NONO*-knockdown cells were transfected with NonO-expressing plasmid (compare Figure 5B, bars III with IV of each gene), suggesting that NonO has a direct effect on the regulation of genes containing the CRE-responsive element. pGST-Rasd1 was transfected in *NONO*-knockdown HEK293T cells, and the presence of Rasd1 had no effect on the transcript levels of the CREB target genes (compare Figure 5B, bars III with V). These results suggest that Rasd1 requires NonO to regulate the transcription of CREB target genes. Next, we investigated the influence of over-expression of Rasd1 and NonO on the transcriptional regulation of *NR4A1*, *FOS* and *NR4A2* in *NONO*-knockdown cells. A similar trend to the results in Figure 5A, bar IV, was observed upon co-transfection of Rasd1- and NonO-expressing plasmids in *NONO*-knockdown cells (Figure 5B, bar VI of *NR4A1* and *NR4A2*). Expression levels of *FOS* remained unaffected by the presence of Rasd1 (compare Figure 5B, bars VI of *FOS* with *NR4A1* and *NR4A2*). Hence, Rasd1 and NonO cooperate to regulate a subset of CREB target genes including *NR4A1* and *NR4A2*.

### Physical presence of Rasd1 and NonO at the CRE-site of *NR4A2* promoter is required for repression of *NR4A2* transcription

ChIP was carried out using forskolin-induced HEK293T lysates transfected with pGST-Rasd1. Anti-NONO was incubated with the lysates, and PCR was performed on the chromatin co-immunoprecipitated along with NONO. Compared to cells transfected with pGST (negative control), there was an increase in the amount of NONO bound to the CRE-site of the *NR4A2* promoter only in cell lysates transfected with pGST-Rasd1 (compare Figure 5C, Lanes 2 and 3). A similar experiment was conducted on HEK293T cells transfected with either pHis-Rasd1 or pHis-Del-NLS (mutant that does not interact with NonO to serve as negative control) to determine if increased binding of NONO to the *NR4A2* promoter could be due to the presence of Rasd1 at the target promoter. Neither Rasd1 nor the mutant, Del-NLS, was observed to be at the CRE-site of the *FOS* promoter (Figure 5D, Lanes 5 and 6). This seemed reasonable, as Rasd1 was unable to work with NonO to suppress the transcription of *FOS*. In the case of the *NR4A2* promoter, only Rasd1 was able to co-immunoprecipitate the *NR4A2* promoter (Figure 5D, Lane 2). The results suggest that binding of Rasd1and NonO to the CRE-site of the *NR4A2* promoter is required for the repression of its transcription.

## Discussion

In this study, we have identified NonO as a novel binding partner of Rasd1 via *in vitro* affinity-based assay, and this interaction is validated using pulldown and co-immunoprecipitation assays. We then studied the roles of Rasd1 and NonO in the cAMP pathway. Our findings show that co-localisation of Rasd1 and NonO in the nucleus is associated with the repression of a subset of CREB target genes. This process involves the GTP hydrolysis activity of Rasd1 and requires interaction of Rasd1 with full-length NonO at the CRE-site of the target promoter. We propose that Rasd1 modulates the function of NonO to down-regulate CREB target genes, *NR4A1* and *NR4A2*.

Our results show that deletion of the putative bipartite nuclear localisation sequence located at the C-terminal portion of Rasd1 does not deter Rasd1 from entering the nucleus, implying that Rasd1 enters the nucleus by other means. Small molecular weight proteins of less than 60 kDa or 9 nm in diameter are able to enter the nucleus via the nuclear pore complex by passive diffusion [37], which is one possible mechanism employed by Rasd1, whose molecular weight is 32 kDa, to enter the nucleus. NonO contains a bipartite nuclear localisation signal, and Rasd1 may bind to NonO to facilitate its entry into the nucleus. Our results indicated that the lack of NonO's NLS prevented accumulation of Rasd1 in the nucleus, which suggests that NonO may play a role in retaining Rasd1 in the nucleus. In addition, studies using Rasd1 mutants show that GDP-bound Rasd1 resulted in cytoplasmic localisation of NonO. This implies that nuclear retention of Rasd1 by NonO is energy dependent and requires GTP-bound Rasd1. Further studies will be required to decipher the exact mechanism employed by NonO to enable the enhanced nuclear presence of Rasd1. Recently, Chuderland *et al.* identified a novel nuclear localisation sequence termed the NTS (nuclear translocation sequence), composed of phosphorylated S/T-P-S/T, that enables nuclear translocation of the protein via binding to importin 7 [38], [39]. This mode of nuclear translocation is used by proteins of different signaling pathways, including the ERK (extracellular signal-regulated kinase) pathway, and was initially discovered on ERK-2 [38], [39]. This mode of nuclear entry enables rapid response to signaling and also adequately explains the increase in the amount of Rasd1 found in the nucleus in the presence of NonO. Interestingly, a ‘TPT’ amino acid sequence is found in Rasd1; this sequence is evolutionarily conserved, and is also present in Rasd2, a paralog of Rasd1. Further studies are required to determine if nuclear translocation of Rasd1 requires its ‘TPT’ sequence.

In our study, we observed that NonO only upregulates CREB-GAL4DBD fusion protein activity slightly via reporter gene assay. This observation contradicts a previous study, which suggests that NonO works as a strong co-activator of the cAMP-dependent pathway via interaction with TORC2, tranducers of the regulated CREB [13]. In our reporter gene system, the CREB-GAL4DBD fusion construct used in the assay lacks the bZIP domain. It has been shown that TORC2 interacts with CREB via the bZIP domain [40]. Hence, deletion of the bZIP domain in the CREB-GAL4DBD construct prevents interaction between CREB and TORC2 [8]. This may serve to explain the minor induction of the luciferase activity upon transfection of NonO in HEK293T cells.

Many transcription factors, RNA-binding proteins and transcriptional co-regulators are known to be bi- or multi-functional proteins. Some bi-functional proteins, including CoAA, PGC-1, CAPERα and CAPERβ, and steroid receptors, are shown to be involved in transcription co-activation and alternative splicing [15], [41], [42], [43]. Multi-functional proteins like NONO and PSF (polypyrimidine tract-binding protein-associated splicing factor) perform RNA processing functions, transcriptional activation and repression, and RNA transport [12], [13], [14], [15], [31], [32], [33], [44], [45]. In addition, NONO is also involved in circadian rhythm as an antagonist of Per1 [46]. Multiple lines of evidence indicate that NonO is a multi-tasking protein with bimodal function in transcription [12], [13], [14], [15], [31], [32], [33], [46]. NonO is known to serve as a co-activator by interacting with TORC2 to up-regulate target genes of the cAMP-dependent pathway [13]. However, NonO is also known to repress transcription by recruiting histone deacetylase (HDAC) to the target promoter by itself or via interaction with PSF [12], [15]. In addition, NonO is known to interact directly with histone to suppress transcription of prolyl-4-hydroxylase α1 upon induction of cells with TNFα [15]. Moreover, NonO has been shown to serve as both co-activator and repressor of androgen receptor-regulated gene transcription depending on the other proteins associated with the transcriptional initiation complex [44], [45]. Interestingly, another interacting partner of NonO, DJ-1, has been shown to switch NonO from a co-repressor to a co-activator for neuroprotection functions [47].

In this study, we show that binding of Rasd1 to NonO signals NonO to switch from a co-activator to co-repressor mode to suppress transcription of a subset of the CREB target genes. Our case is similar to NonO's regulation of the androgen receptor where NonO can either activate or repress transcription of the androgen receptor depending on the proteins associated with it [44], [45]. Since Rasd1 is known to serve as a transcriptional co-repressor of FE-65 [19], and as an antagonist to the function of transcription factor, Ear-2, in the repression of *Renin*'*s* transcription [34]; it is conceivable that Rasd1 might enable NonO to serve as a transcriptional co-repressor of the CREB signaling pathway. We observed that co-transfection of pNonO-V5 and pGST-Rasd1 resulted in a substantial increase in the nuclear localisation of Rasd1. This finding resembles that of Lau *et al* [19], where co-transfection of Rasd1 and FE-65 results in an increased nuclear distribution of Rasd1, and suggests that nuclear translocation of Rasd1 is required for suppression of target-gene transcription. In addition, we observed that Rasd1 and NonO co-suppress the transcription of a subset of CREB target genes. It is intriguing that, unlike *NR4A1* and *NR4A2* transcripts, *FOS* transcription was unaffected by Rasd1 and NonO. The discrepancy observed might be attributed to the differences in the mechanisms involved in the transcriptional regulation of *NR4A* and *FOS*. Transcriptional regulation of *FOS* takes place at both transcriptional initiation and elongation processes, which allows an additional level of control of the *FOS* gene [48], [49], [50]. Currently, *NR4A* proteins are only known to be regulated by transcription factors at the transcription initiation stage [13], [51], [52]. Our study also suggests that Rasd1 and NonO bind the CRE-site of the *NR4A2* promoter to repress its transcription and that the mechanism employed by Rasd1 and NonO in the repression of transcription of *NR4A* genes involves the regulation of proteins required for the transcription initiation step. Further studies will need to be performed to explore the mechanism involved.

Many signal transduction pathways converge in the nucleus through modulating CREB, whose phosphorylation pattern influences binding of its co-activators, including CBP/p300 in the presence or absence of TORC2 [7], [8]. Phosphorylation of CREB by PKA and other kinases of the cAMP signaling pathway activates CREB to recruit co-activators, other transcription factors, and general transcription factors to the target promoter, resulting in the transactivation of target genes [13]. It remains unclear how CREB is able to converge diverse signals and elicit differential effects on target gene expression. In the case of the cAMP-dependent pathway, the co-regulators that interact with CREB may play an important role for the cell to have a specific response in different contexts [30]. There are a plethora of CREB co-regulators, and their activities are known to be regulated by different mechanisms. These include sequestration of the co-activator, TORC, that is normally anchored in the cytoplasm by 14-3-3 proteins in the absence of stimulation of the pathway [7], [8]; and competition for limiting factor such as CBP, which is a co-activator required for multiple signaling pathways [9]. In this study, our findings lend credence to a new mode of regulation of co-activators of the cAMP pathway – modulation of co-activator function. We define modulation in the context whereby the identity of an interacting partner of the co-activator determines the role of the co-activator in regulation of the target gene upon induction of the cAMP pathway.

*NR4A1* and *NR4A2* are clock-controlled genes oscillating in multiple tissues [53], [54] and are CREB-target genes whose expressions are up-regulated upon activation of the cAMP pathway [13]. The nuclear orphan receptors 4A (NR4A) subgroup belongs to the nuclear hormone receptor family and consists of transcription factors capable of recognising the NGFI-B response element (NBRE) [55], [56], [57], [58]. NR4A proteins can bind to DNA as monomers, homodimers and heterodimers [59], [60]. Transcription factor NR4A2 is an immediate early gene induced by many external stimuli, including retinoic acid, forskolin, prostaglandin E_2_, and dexamethasone [51], [56], [61], [62]. NR4A proteins play important roles in metabolism and in the pathogenesis of various diseases including colorectal cancer, Alzheimer's disease, familial Parkinson's disease, schizophrenia, inflammatory arthritis, and manic depression [51], [56], [61], [63], [64], [65], [66]. In addition, NR4A proteins also play a crucial part in CREB-dependent neuro-protection, cell survival and cell transformation of HeLa cells [52], [63], [67], [68]. Expression of NR4A proteins is up-regulated by the cAMP pathway for initiation of the survival of HeLa cancer cells [68]. NR4A proteins are activated via the cAMP pathway through PGE2 in human colorectal cancer cells [51]. HeLa cells with reduced levels of NR4A proteins displayed a higher tendency for cell death through anoikis [68].

Circadian rhythm is an endogenous 24-hour cycle consisting of an input pathway, master clock, and an output pathway; the underlying mechanism of rhythmistic control is conserved across species [69]. The clock regulates biological processes in a temporal manner by synchronising peripheral oscillators possibly through glucocorticoids, enabling the adaptation and synchronisation of hormones, sleep-wake cycles, and daily activities with changing environmental cues [69], [70], [71]. Recent findings have linked nutrient and energy metabolism to circadian rhythm [54], [72]. Genes involved in metabolism such as NR4A family are known to oscillate in liver and muscle [54]. The circadian rhythm can be modulated by external signals (light, food, temperature), and these signals are conveyed through the MAPK and cAMP-signaling pathways [70], [73]. Interestingly, central and peripheral oscillators are sensitive to entrainment by light (photic) and food (non-photic), respectively [73], [74], [75]. The phase-resetting signals provided through food on peripheral clocks are inhibited by glucocorticoids [76].

Interestingly, expression of *NR4A2* and *Rasd1* are known to be repressed and upregulated by glucocorticoids, respectively [17], [61]. Evidence provided by Rasd1 knockout mice show that Rasd1 may be involved in the input pathway of the circadian rhythm by enhancing photic response and reducing the stimulus provided from non-photic inputs [77]. This suggests that Rasd1 might function as the bridging molecule for glucocorticoids to inhibit the phase-resetting pulses by food on peripheral clocks. Rasd1 may then work with NonO to repress genes involved in metabolism activated via the cAMP pathway, which results in selective repression of a subset of target genes. Hence, modulation of *NR4A1* and *NR4A2* expression by Rasd1 and NonO could have a major impact on the circadian control, and disruption of this process can give rise to metabolic diseases and cancer development [78], [79], [80], [81].

## Materials and Methods

### Plasmid constructs

For information on all primer sequences used for cloning, please refer to Table S1. Coding sequence of mouse *Rasd1* (843 bp) was amplified from mouse brain cDNA library PACT2 and cloned in frame via restriction sites *Kpn*I and *Xho*I into expression vectors – pcDNA4/HisMax^©^B (V864-20, Invitrogen, USA, CA), and pXJGST vector modified from parent plasmid pXJ FLAG [82] by replacing the FLAG coding sequence with the GST coding sequence – and designated as pHis-Rasd1 and pGST-Rasd1, respectively. pHA-Rasd1 was constructed by insertion of a HA-tag at the 3′ end of the coding sequence of *Rasd1*and cloned into pcDNA4/HisMax^©^B via *Kpn*I and *Xho*I. Mouse clone of *NonO* (1.4 kb) and its truncated constructs were amplified from MGC-6432 (ATCC, USA, VA), and cloned in frame via restriction sites *Kpn*I and *Xho*I into pcDNA3.1/V5-His B (V810-20, Invitrogen, USA, CA) and pXJGST vectors. PCR-based, site-directed mutagenesis was used to construct all other mutants of NonO and Rasd1, which were cloned into pcDNA3.1/V5-His B, pXJGST and pcDNA4/HisMax^©^B vectors. For *NONO* knockdown studies, *NONO*-shRNA was constructed by cloning of the oligonucleotide that targets mRNA of *NONO* into pSUPER.puro (VEC-PBS-0007/0008, OligoEngine, USA, WA) vector. The negative control, Neg-shRNA, was constructed by jumbling up the sequence of the oligonucleotide that was used for cloning of *NONO*-shRNA. Annealed oligonucleotides were cloned in pSUPER.puro via *Bgl*II and *Hin*dIII sites. BLAST was performed to ensure specificity of *NONO*-shRNA, and that Neg-shRNA did not target any non-specific sequences. The annealing process was performed in the annealing buffer (100mM NaCl and 50mM Hepes, pH 7.4) in BioRad PCR machine: 90°C for 4min, 70°C for 10min, and ramped to 37°C over a period of 45min, kept constant at 37°C for 15min, and ramped to 10°C over a period of 45min.

### Cell culture and transient transfection

HEK293T (ATCC CRL-11268), PC-12 (ATCC CRL-1721), and COS-7 (ATCC CRL-1651) cells were cultured as previously described [26], [83]. All transfections were performed the next day after seeding of 3×10^5^ cells into each well of a 6-well plate unless stated otherwise. COS-7 cells were transfected using Lipofectamine™ 2000 (Invitrogen, USA, CA) according to manufacturer's instructions. Calcium phosphate method was employed for transfection of plasmids into HEK293T cells.

### *In vitro* pulldown assay and mass spectrometry

*In vitro* affinity pulldown assay using mammalian cell lysates was conducted to identify novel interacting partners of Rasd1. COS-7 cells seeded on 10cm plates were transfected the next day with His-Rasd1 (24 µg) or pcDNA4/HisMax^©^B (24 µg) (negative control) at 90% confluence. Cells were harvested as previously stated [84]. 20 µl of Ni-NTA magnetic agarose beads (Qiagen, USA, CA) were added to purify His-Rasd1 for 1 hour at 4°C. Beads were washed according to manufacturer's instructions. This was followed by incubation of His-Rasd1-bound beads with PC-12 lysates overnight (O/N) at 4°C. After incubation, beads were washed and bound-proteins were eluted by heating in Laemmli buffer at 95°C for 10 minutes. Samples were fractionated on 12% SDS-PAGE acrylamide gel and subsequently stained with Coomassie blue. Bands of interest were excised by scalpels and cut into 1mm^3^ cubes. The cubes were then destained with 50% methanol at room temperature before digestion was carried out with 10 ng/µl Trypsin (Promega, USA, WI) O/N at 37°C. Subsequently, 50% ACN/5% Trifluoroacetic acid was used to extract peptides. The peptides were then dried under vacuum and cleaned with ZipTip® C_18_ (Merck Millipore, USA, MA) according to manufacturer's instruction. MALDI-TOF/MS was used to elucidate the identity of the unknown bands. SwissProt database was used for the analysis of the peptide spectra obtained. The MALDI/TOF-MS score obtained for NonO was 199.

### GST pulldown *in vivo* interaction studies

COS-7 cells were co-transfected with plasmids expressing NonO (2 µg) and Rasd1 (2 µg). Cells were harvested as previously stated [84]. Interaction studies were performed using MagneGST™ particles (Promega, USA, WI) according to manufacturer's instructions. Bound proteins were eluted by heating in Laemmli buffer followed by SDS-PAGE and Western blotting.

### Co-immunoprecipitation (co-IP) assay

Co-IP of endogenous proteins was performed by scraping one 10 cm plate of HEK293T cells in 1.5 ml of PBS, followed by centrifugation at 13,000 rpm for 1 minute. The cells were lysed with NP40 lysis buffer (1% NP40, 150 mM NaCl, 50 mM Tris-Cl, pH 8.0, 1% deoxycholic acid, 0.1% SDS, protease inhibitor (Roche, Switzerland, Basel)) and incubated at 4°C for 20 minutes. The crude lysate was cleared by centrifugation at 13,000 rpm for 20 minutes at 4°C. Experiment was performed with rProtG agarose beads (Invitrogen, USA, CA) as previously described [84]. The pre-cleared lysates were incubated with rabbit polyclonal anti-NMT55/p54NRB IgG (Abcam, UK, Cambridge) or rabbit control IgG (Abcam, UK, Cambridge; negative control). Co-IP using mouse brain lysates was performed using goat polyclonal anti-Rasd1 (Abcam, UK, Cambridge) as described elsewhere [84]. NonO was detected using goat anti-NONO (Abcam, UK, Cambridge) or rabbit anti-NONO (Santa Cruz, USA, CA).

### Reporter gene assay

Effects of Rasd1 and NonO in the CREB signaling pathway were investigated using PathDetect CREB trans-Reporting System (Stratagene, USA, CA). PathDetect CREB trans-Reporting System is a GAL4-dependent reporter gene assay. Factors influencing the phosphorylation of CREB protein (fused to GAL4-DNA-binding domain) will be monitored effectively by similar changes in the luciferase activity. HEK293T cells were transfected with pSV-β-Gal (β-Galactosidase; internal reporter), pFR-Luc (reporter plasmid with 5xGAL4 binding site and TATA box as minimal promoter), and pFA2-CREB (CREB (1–280) fused to GAL4-DNA binding domain (dbd) trans-activator plasmid) plus different combinations of pHis-Rasd1, and pNonO-V5 as indicated in Figure 2. pcDNA3.1/V5-His B and pcDNA4/HisMax^©^B served as negative controls for pNonO-V5 and pHis-Rasd1, respectively. Cells were induced with 20 µM forskolin (Sigma-Aldrich, USA, MO) for 4 hours before harvesting. Luciferase assay was performed using Luciferase Assay System (Promega, USA, WI) according to manufacturer's protocol. 20/20^n^ Luminometer (Promega, USA, WI) was used to measure luciferase levels. β-gal levels were measured using β-Gal Enzyme Assay System (Promega, USA, WI) according to manufacturer's protocol. Normalisation of data was performed using luciferase values of wells transfected with pFC2-dbd (negative control) construct containing only GAL4-dbd and with no trans-activator function. All values of empty vectors were set as 1. Statistical analyses were performed using two-tailed unpaired student's t-test.

### Indirect immunofluorescence

HEK293T and COS-7 cells were transfected as indicated in Figures 3 and 4, respectively. Experiment was performed as described previously [19]. Primary antibody incubation was performed with mouse anti-V5 or anti-Xpress (1∶250; Invitrogen, USA, CA) and GST-Alexa Fluor 488nm (1∶100; Santa Cruz, USA, CA). After that, samples were incubated with secondary antibody, goat anti-mouse-Alexa Fluor 568nm (1∶200; Sigma-Aldrich, USA, MO). Cells were subjected to Zeiss LSM510 META confocal microscopy studies.

### Reverse transcription and Real time PCR

iScript™ cDNA synthesis kit (Bio-Rad, USA, CA) was performed according to manufacturer's instructions. Quantitative Real time PCR was carried out using iTaq™ SYBR® Green Supermix with ROX (Bio-Rad, USA, CA) according to manufacturer's instructions. Primer sequences used for Real time PCR were listed in Table S2.

### Chromatin immunoprecipitation (ChIP)

Confluent HEK293T cells cultured in 10 cm plates were cross-linked with Formaldehyde (37% stock) for 15min at 37°C. The reaction was quenched with 1M glycine for 5min at RT. ChIP was performed using Protein A agarose/Salmon sperm DNA beads according to manufacturer's instructions (Merck Millipore, USA, MA). Appropriate antibody (2 µg) was added to the pre-cleared lysates and incubated O/N at 4°C on a rotating platform. This was followed by phenol-chloroform extraction, and subsequent PCR was conducted using the relevant primers to study target of interests (refer to Table S2 and Figure 5, C and D).

## Supporting Information

Figure S1 Study of gene expression induced by forskolin in HEK293T cells. Real time PCR was performed as stated in Figure 5. Only NR4A1, NRR4A2 and FOS transcripts are upregulated upon treatment with forskolin (Bars VIII, X and XIV). β-actin was used as an internal control for normalization.
(TIF)

Table S1 Primer sequences used for construction of vectors for protein expression and knockdown studies.
(TIF)

Table S2 Primer sequences used for Real time PCR and ChIP experiments.
(TIF)
