# Supplementary figures and images for: Rasd1 Modulates the Coactivator Function of NonO in the Cyclic AMP Pathway
Source: PLoS One. 2011 Sep 7;6(9):e24401. doi: 10.1371/journal.pone.0024401 (PMC3168489; doi:10.1371/journal.pone.0024401)

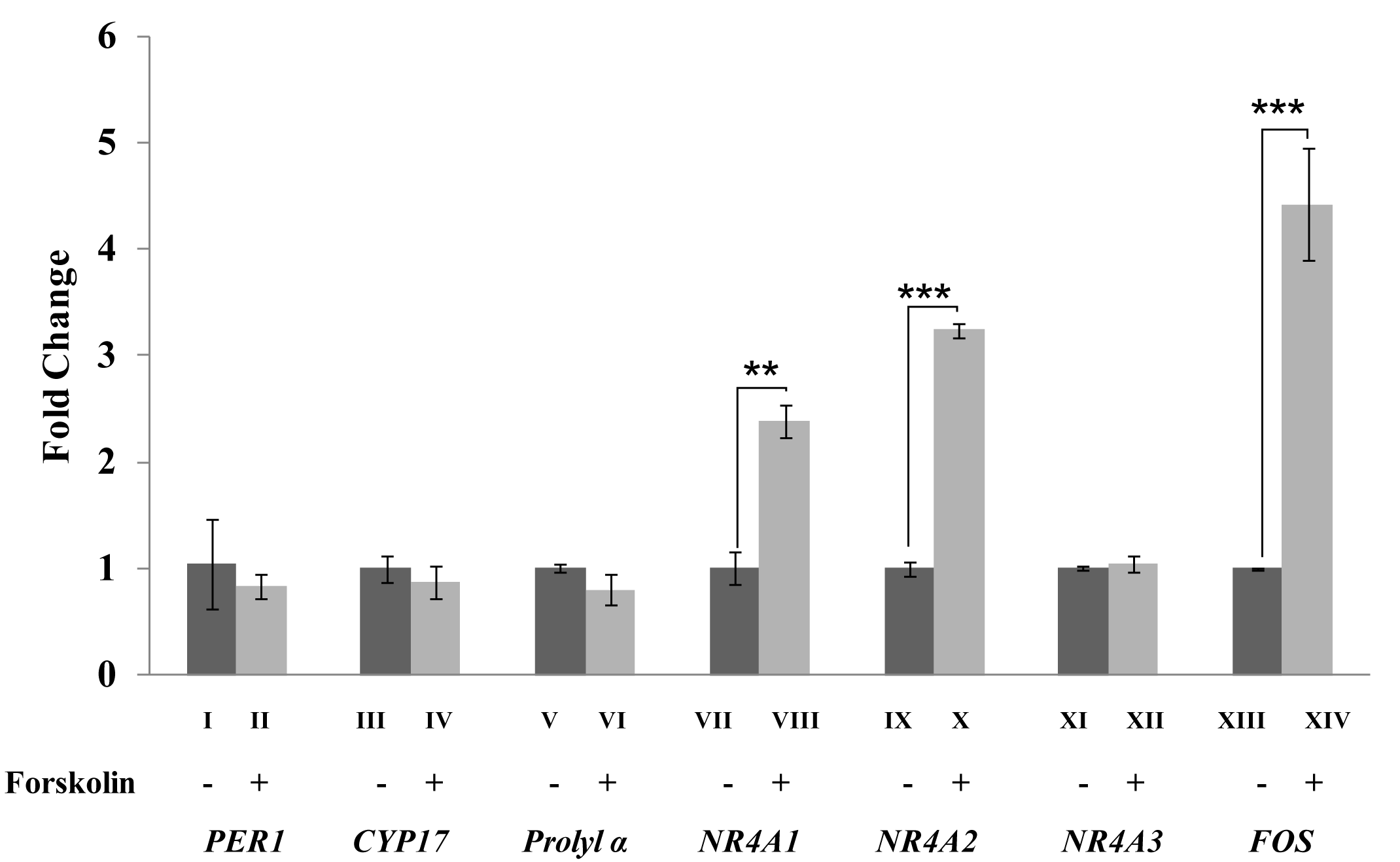

Supplement: Figure S1 — Study of gene expression induced by forskolin in HEK293T cells. Real time PCR was performed as stated in Figure 5. Only NR4A1, NRR4A2 and FOS transcripts are upregulated upon treatment with forskolin (Bars VIII, X and XIV). β-actin was used as an internal control for normalization. (TIF) [file pone.0024401.s001.tif]
